# Birth weight influences cardiac structure, function and disease risk: evidence of a causal association

**DOI:** 10.1093/eurheartj/ehad631

**Published:** 2023-09-20

**Authors:** Maddalena Ardissino, Alec P. Morley, Eric A W Slob, Art Schuermans, Bilal Rayes, Zahra Raisi-Estabragh, Antonio de Marvao, Stephen Burgess, Tormod Rogne, Michael C Honigberg, Fu Siong Ng

**Affiliations:** 1 National Heart and Lung Institute, Imperial College London, UK; 2 Department of Medicine, School of Clinical Medicine, University of Cambridge, UK; 3 Medical Research Council Biostatistics Unit, University of Cambridge, UK; 4 Department of Applied Economics, Erasmus School of Economics, Erasmus University Rotterdam, the Netherlands; 5 Erasmus University Rotterdam Institute for Behavior and Biology, Erasmus University Rotterdam, the Netherlands; 6 Erasmus School of Social and Behavioural Sciences, Erasmus University Rotterdam, the Netherlands; 7 Department of Cardiovascular Sciences, KU Leuven, Leuven, Flanders, Belgium; 8 Program in Medical and Population Genetics, Broad Institute of Harvard and MIT, Cambridge, MA, USA; 9 Cardiovascular Research Center, Massachusetts General Hospital, Harvard Medical School, Boston, MA, USA; 10 William Harvey Research Institute, NIHR Barts Biomedical Research Centre, Queen Mary University of London, UK; 11 Barts Heart Centre, St Bartholomew’s Hospital, Barts Health NHS Trust, UK; 12 Department of Women and Children’s Health, King’s College London, United Kingdom; 13 British Heart Foundation Centre of Research Excellence, School of Cardiovascular Medicine and Sciences, King’s College London, United Kingdom; 14 Medical Research Council, London Institute of Medical Sciences, Imperial College London, UK; 15 Cardiovascular Epidemiology Unit, Department of Public Health and Primary Care, University of Cambridge, UK; 16 Department of Chronic Disease Epidemiology, Yale School of Public Health, USA; 17 Cardiology Division, Department of Medicine, Massachusetts General Hospital, Boston, MA, USA

**Keywords:** cardiac MRI, birth weight, mendelian randomization, genetic effects, maternal, fetal, intrauterine

## Abstract

**Background and Aims:**

Low birth weight is a common pregnancy complication, which has been associated with higher risk of cardiometabolic disease in later life. Prior Mendelian randomization (MR) studies exploring this question do not distinguish the mechanistic contributions of variants that directly influence birth weight through the fetal genome (direct fetal effects), *versus* variants influencing birth weight indirectly by causing an adverse intrauterine environment (indirect maternal effects). In this study, MR was used to assess whether birth weight, independent of intrauterine influences, is associated with cardiovascular disease risk and measures of adverse cardiac structure and function.

**Methods:**

Uncorrelated (r^2^<0.001), genome-wide significant (p<5×10^−8^) SNPs were extracted from genome-wide association studies (GWAS) summary statistics for birth weight overall, and after isolating direct fetal effects only. Inverse-variance weighted MR was utilised for analyses on outcomes of atrial fibrillation, coronary artery disease, heart failure, ischaemic stroke, and 16 measures of cardiac structure and function. Multiple comparisons were accounted for by Benjamini-Hochberg correction.

**Results:**

Lower genetically-predicted birth weight, isolating direct fetal effects only, was associated with an increased risk of coronary artery disease (OR 1.21 [1.06 to 1.4137 p=0.031), smaller chamber volumes, lower stroke volume, but higher contractility.

**Conclusions:**

The results of this study support a causal role of low birth weight on cardiovascular disease, even after accounting for the influence of the intrauterine environment. This suggests that individuals with a low birth weight may benefit from early targeted cardiovascular disease prevention strategies, independent of whether this was linked to an adverse intrauterine environment during gestation.

## Introduction

Low birth weight is a common pregnancy complication, affecting approximately 15% of live births globally^1^. Multiple observational studies have described that low birth weight is associated with higher risk of cardiometabolic disease in later life^2–5^. These findings have given rise to the Developmental Origins of Health and Disease (DOHaD) hypothesis, suggesting that adverse intrauterine environment and nutritional deprivation during fetal growth promote a series of metabolic adaptations that ultimately foster the development of cardiovascular disease^6^. The majority of data supporting this hypothesis is derived from observational studies^2–5^. However, in the observational setting, it is difficult to definitively establish that this relationship is causal. It is well recognised both birth weight and cardiovascular disease risk are strongly influenced by many notoriously difficult-to-measure economic and socio-behavioural factors, almost certainly contributing to a degree of residual confounding.

Mendelian randomization (MR) is a genetic epidemiological method that leverages the random process of allele assortment at conception, which leads to an effective ‘randomization’ of individuals to high or low genetic risk of a phenotype such as low birth weight, to help establish causality^7^. This effective randomization limits the liability to influence by reverse causation and confounding and can therefore provide evidence to support a causal association between the exposure and outcome in question^8,9^. In previous MR studies, lower birth weight has been associated with higher risk of coronary artery disease^10–12^; but conversely a lower risk of atrial fibrillation^13^.

Though these studies address the issue of observational confounding and suggest a causal role supporting the DOHaD hypothesis, they are limited by the lack of adjustment for the expected correlation between maternal and fetal phenotypes. Indeed, in a genome-wide association study (GWAS) on individuals’ birth weight, the genetic effects will include a mixture of (1) genetic variants that directly influence birth weight through the fetal genome, (2) maternal genetic variants that promote an adverse intrauterine environment, which will be correlated with fetal genotype (r=~0.5, due to direct inheritance during conception) and (3) paternal effects, which have been shown to be negligible^14^. All available MR studies to date have not differentiated these effects^10–13^, except for one study exploring cardiovascular risk factors but not outcomes^15^. For this reason, currently available data does not provide any mechanistic information about whether the association is driven by direct effects of birth weight, by an adverse intrauterine environment, or both.

Recently, two GWAS studies have specifically isolated the variants that exert genetic effects through the fetal genome from those related to adverse intrauterine environments. First, Warrington *et al.* utilised structural equation modelling to adjust for indirect maternal influences in the genetic effects on birth weight, thus isolating the direct fetal effects only in a GWAS in participants of the Early Growth Genetics (EGG) Consortium and the UK Biobank (UKB)^14^. More recently, Juliusdottir *et al.* used a clustering-based method to identify variants affecting birth weight through the fetal genome only, separating these from variants influencing birth weight through maternal or paternal genomes, in the Icelandic birth register cohort^16^. To date, two investigations have adopted the former study in MR analyses. These were aimed at assessing associations with cardiometabolic risk factors, and both studies identified that lower birth weight, even after isolating direct fetal effects only, was associated with worse cardiometabolic profile^14,15^. However, it is unclear whether this result might extend to overt cardiovascular disease and adverse cardiac remodelling.

In this study, we use large-scale genetic data to explore the association of lower birth weight, overall and after only isolating direct fetal effects, with cardiovascular diseases and with multiple cardiac magnetic resonance imaging (CMR) markers of structure and function. For the associations of low birth weight through direct fetal effects with cardiovascular outcomes, we aim to explore whether traditional cardiovascular risk markers mediate the association, to explore whether intervening on these factors in a clinical setting might mitigate the excess cardiovascular risk conferred by lower birth weight.

## Methods

### Study design

A summary of study data sources is provided in Table 1. The paper is reported on the basis of recommendations by the Strengthening the Reporting of Observational Studies in Epidemiology using Mendelian Randomization (STROBE-MR) Guidelines^17^. All statistical analyses were performed using R v4.1.2^18^ using the TwoSampleMR^19^ and Mendelianrandomization packages^20^. The Graphical Abstract was created with BioRender.com.

### Instrumental variable selection

**Figure 1:**
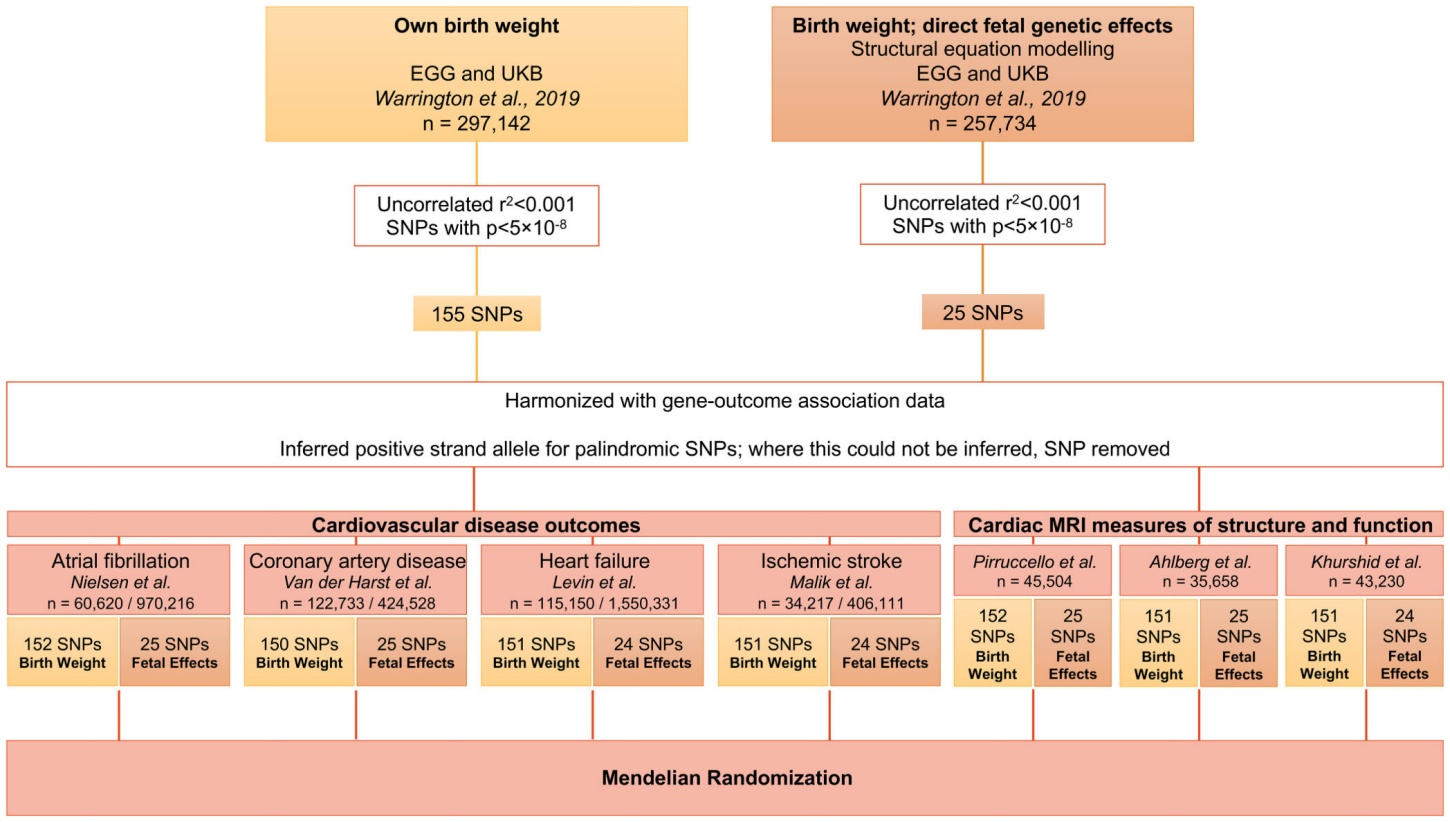
Diagram representing study design and data analysis workflow. EGG = Early Growth Genetics, UKB = UK Biobank, SNP = single nucleotide polymorphism.

For primary analysis, instrumental variants were extracted from Warrington *et al.’s* meta-analysis using the Early Growth Genetics (EGG) consortium and UK Biobank^14^. This study included 297,142 individuals with birth weight data, 257,753 of whom were used in a structural equation modelling approach to adjust the overall genetic association estimates for indirect maternal genetic influences, thus producing genetic association estimates that isolate only the direct fetal genetic effects. The methods are described at the original publication^21^. The measure of birth weight was z-transformed; and all models were adjusted for sex, gestational duration and the first four ancestry-informative principal components (to capture population stratification). From the summary statistics of this study, genome-wide significant (p<5×10^−8^) uncorrelated (r^2^<0.001) variants were extracted as instrumental variables for the exposures of (1) birth weight overall measured in standard deviations, 155 SNPs, and (2) direct fetal genetic influence on birth weight, 25 SNPs. The analysis flowchart is depicted in Figure 1.

A replication analysis was performed utilising data from Juliusdottir *et al.’s* investigation which utilised a different methodology^16^. In this study, a meta-analysis GWAS on own birth weight was performed including 423,683 individuals from the Iceland Birth Register, EGG consortium, and UKB. For own birth weight, 199 uncorrelated (r^2^<0.001) genome-wide significant (p<5×10^−8^) instrumental variants were extracted from summary statistics of this analysis. Then, using phased genotype data of 104,920 parent-offspring trios, the authors analysed the maternal transmitted alleles, paternal transmitted alleles, and maternal non-transmitted alleles separately, formally testing which combination of alleles best explained the association with birth weight. Based on these results, the variants influencing birth weight were clustered using Gaussian mixture model–based clustering into eight separate clusters, based on the pattern of effect. Further description of the methods is available in the original publication. Among these, clusters 1, 2 and 3 included variants affecting birth weight through the fetal genome only. In the present study, the 80 uncorrelated (r^2^<0.001) genome-wide significant (p<5×10^−8^) instrumental variants in these three clusters were extracted for the analysis of direct fetal effects on birth weight. The flowchart for instrumental variable selection for the sensitivity analysis is depicted in Supplementary Figure 1.

Instrument strength was quantified using F-statistics. The F-statistic measures the ratio of the mean square of the model to the mean square of the error. F-statistic for univariable analyses was calculated using the formula

$$F=\frac{\left(n-k-1\right)}{k}\frac{\left(R^{2}\right)}{\left(1-R^{2}\right)}$$

where *R*^2^ is the explained variance in the regression of all SNPs, *n* is the number of participants in the study, k is the number of instrumental variants . The *R*^2^ was calculated as the sum of SNP-wise *R*^2^ of instruments, which is obtained as follows

$$R^{2}=\frac{F}{\left(n-2+F\right)}\;\text{with}\;F={\left(\frac{\beta }{SE(\beta )}\right)}^{2}$$

where β represents the effect size of the genetic variant in the exposure GWAS, and SE(β) represents the standard error of the effect size of the genetic variant in the exposure GWAS. For multivariable analyses, instrument strength was assessed using conditional F-statistics calculated using the MVMR package^22,23^.

### Study outcomes

Genetic association estimates for the cardiovascular outcomes were extracted from GWAS summary statistics for atrial fibrillation (60,620 cases and 970,216 controls)^24^, coronary artery disease (122,733 cases and 424,528 controls)^25^, heart failure (115,150 cases and 1,550,331 controls)^26^ and ischaemic stroke (34,217 cases and 406,111 controls)^27^.

Genetic association estimates for the majority of CMR parameters were extracted from publicly available GWAS summary statistics of Pirruccello *et al.’s* recent study in 45,504 participants in the UK Biobank Imaging Cohort^28^. The imaging outcomes considered included: left ventricular end systolic volume (LVESV), left ventricular end diastolic volume (LVEDV), left ventricular stroke volume (LVSV), left ventricular ejection fraction (LVEF), right atrial maximum area (RA Max), right atrial minimum area (RA Min), right atrial fractional area change (RA FAC), right ventricular end systolic volume (RVESV), right ventricular end diastolic volume (RVEDV), right ventricular systolic volume (RVSV), right ventricular ejection fraction (RVEF), proximal pulmonary artery diameter (Prox PA Diam), ascending aorta diameter (Asc Aorta Diam). Genetic association estimates for left atrial maximum volume (LA Max) and left atrial total ejection fraction (LATEF) were derived from Ahlberg *et al.’s* study on 35,658 individuals from the UK Biobank Imaging Cohort^29^. Genetic association estimates for left ventricular mass (LV Mass) was derived from Khurshid *et al.’s* study on 43,230 participants of the UK Biobank Imaging Cohort^30^. CMR outcomes were selected based on the availability of large cohort GWAS summary statistics. All outcomes were indexed by body surface area; except LATEF, LVEF, RA FAC and RVEF, which are dimensionless.

### Mendelian randomisation

Gene-exposure association data was individually harmonised with gene-outcome association using the TwoSampleMR package in R. During harmonisation, the positive strand allele was inferred where this was possible, and where this was not possible the SNP was not used in further analysis. Only SNPs with gene-exposure and gene-outcome association data present were used in the analysis.

Inverse-variance weighted (IVW) MR with multiplicative random effects was used as the primary analysis method to estimate the association between genetically-predicted birth weight (overall, and after isolating the direct fetal genetic effects) and each outcome^31^.

For cardiovascular outcomes, results are presented as odds ratios (OR) with respective 95% confidence intervals (CI). For the CMR measures, which are continuous, results are presented as coefficients (β) and 95% CI. All presented P-values are adjusted for multiple comparisons using Benjamini-Hochberg correction for a false-discovery rate (FDR) of 5% based the number of tests performed for each exposure.

### Sensitivity analyses

Sensitivity analyses were carried out using weighted median MR and MR-Egger. One of the core assumptions of the IVW MR approach is that genetic instruments are only associated with the outcome through the studied exposure. If genetic variants act through additional, ‘parallel’ biological pathways, these assumptions are violated due to horizontal pleiotropy. Sensitivity analysis using weighted median MR^32^ and MR-Egger were performed to explore whether this was occurring. The weighted median method has been shown to provide consistent estimates when up to half of SNPs are invalid, or pleiotropic^32^. The MR-Egger method can be used to identify the presence of directional pleiotropy under a weaker assumption that the instrument strength is independent of direct effects (InSIDE assumption)^33^. A significant p-value on MR-Egger intercept test suggests potential presence of directional pleiotropy.

### Mediation analyses

Mediation analysis for any putative direct fetal genetic effects was performed using multivariable Mendelian randomization (MVMR). These were performed only where a significant association was identified in the primary univariable analysis of direct fetal genetic effects. The potential mediators considered were chosen among the phenotypes that have been previously identified to associate with birth weight^14^. These included BMI^34^ (n= 434,794, European ancestry), height^35^ (n= 709,594, European ancestry), SBP^36^ (n= 757,601, European ancestry) and T2DM^37^ (80,154 cases and 853,816 controls, European ancestry). For each individual analysis, the mediators to include were based on biological plausibility of mediation based on a formally tested association between the exposure and mediator, and consistency of this with the direction of association on univariable analysis so as to represent a true potential mediating pathway^38^. For this analysis, we used EGG Consortium / UK Biobank meta-analysis data^14^ for direct fetal effects on birth weight, as these data provide genome-wide estimates adjusted for maternal indirect effects; this is in contrast to Juliusdottir *et al.’s* study^16^ where variants are selected if they have direct fetal effects, but the association estimates are not adjusted.

Multivariable MR was performed to estimate the effect of the exposure on the outcome after accounting for the mediator (reported as an adjusted OR with 95%CI). This was then qualitatively compared to the estimate of association in the main univariable inverse-variance weighted analysis. Substantial attenuation after conditioning by the mediator is taken to suggest the presence of a potential mediating pathway^38^.

## Results

### Birth weight and cardiovascular outcomes

**Figure 2:**
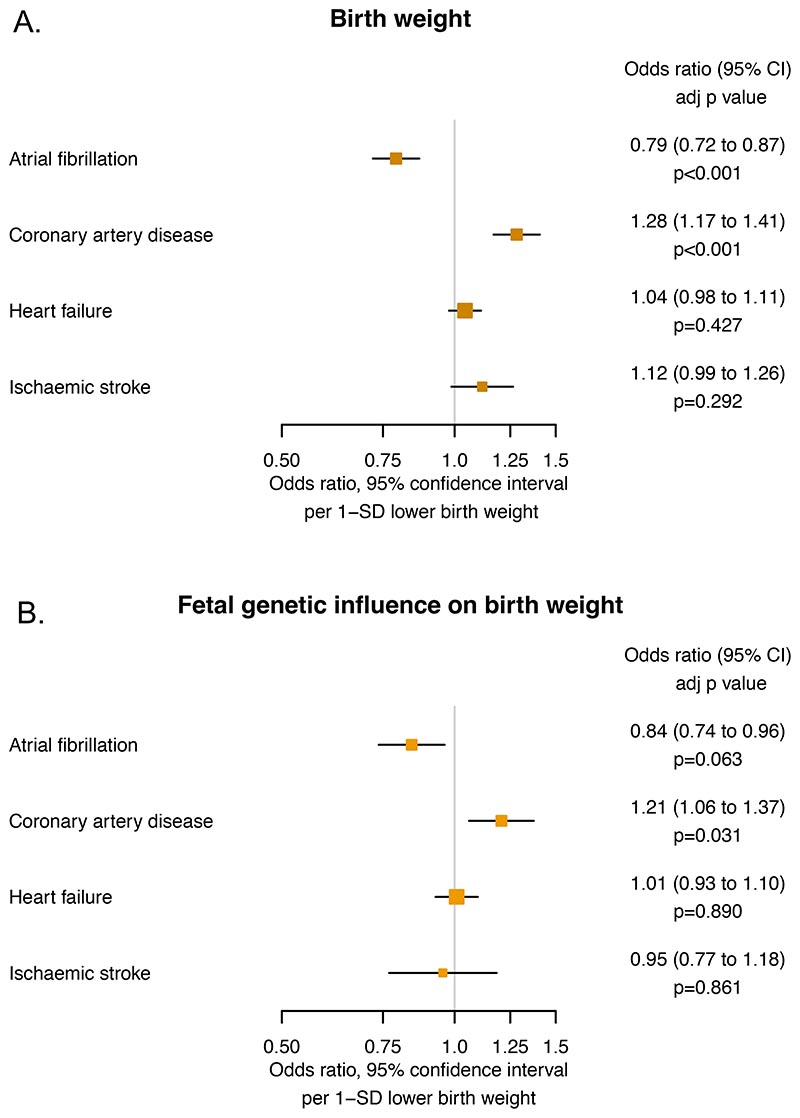
Mendelian randomization estimates for the effects of (A) genetically-predicted birth weight and (B) genetically-predicted birth weight after isolating only variants that act through direct fetal effects, on cardiovascular outcomes. SD = standard deviation.

Lower genetically-predicted birth weight was associated with higher risk of coronary artery disease (OR 1.28 [1.17 to 1.41] p=6.75×10^−6^), but lower risk of atrial fibrillation (OR 0.79 [0.72 to 0.87] p=1.25×10^−6^). There were no statistically significant associations with heart failure (OR 1.04 [0.98 to 1.11] p=0.427) or ischaemic stroke (OR 1.12 [0.99 to 1.26] p=0.292). The results are reported in Figure 2A.

After isolating direct fetal genetic effects, lower birth weight remained associated with higher risk of coronary artery disease (OR 1.21 [1.06 to 1.37] p=3.14×10^−2^). The association with lower risk of atrial fibrillation (OR 0.84 [0.74 to 0.96] p=0.063) remained consistent in direction and magnitude, however, was no longer statistically significant after accounting for multiple testing. There were no statistically significant associations with heart failure (OR 1.01 [0.93 to 1.10] p=0.890) or ischaemic stroke (OR 0.95 [0.77 to 1.18] p=0.861). The results are reported in Figure 2B.

### Birth weight and cardiac structure and function

For cardiac structure and function, all outcomes are presented as the change in the beta value per 1 – SD lower birth weight. Lower genetically-predicted birth weight was associated with lower indexed left chamber volumes: LVESV (β −0.15 [−0.22 to −0.08] p=3.23×10^−4^), LVEDV (β −0.16 [−0.24 to −0.08] p=3.71×10^−4^), LVSV (β −0.12 [−0.19 to −0.04] p=8.53×10^−3^). However, lower birth weight was associated with greater indexed LV mass (β 0.09 [0.02 to 0.17] p=0.022).

Associations with right-sided measures followed a consistent pattern of lower indexed chamber volumes with lower birth weight: RVESV (β −0.18 [−0.25 to −0.11] p=1.35×10^−5^), RVEDV (β −0.17 [−0.25 to −0.10] p=1.05×10^−4^) and RVSV (β −0.12 [−0.19 to −0.04] p=8.53×10^−3^). Additionally, lower birth weight was associated with lower indexed RA areas: including indexed RA Max (β −0.13 [−0.21 to −0.06] p=2.82×10^−3^) and indexed RA Min (β −0.15 [−0.22 to −0.07] p=3.23×10^−4^). However, lower birth weight was associated with higher RA FAC (β 0.08 [0.02 to 0.14] p=0.017) and RVEF (β 0.09 [0.03 to 0.15] p=9.22×10^−3^)

**Figure 3:**
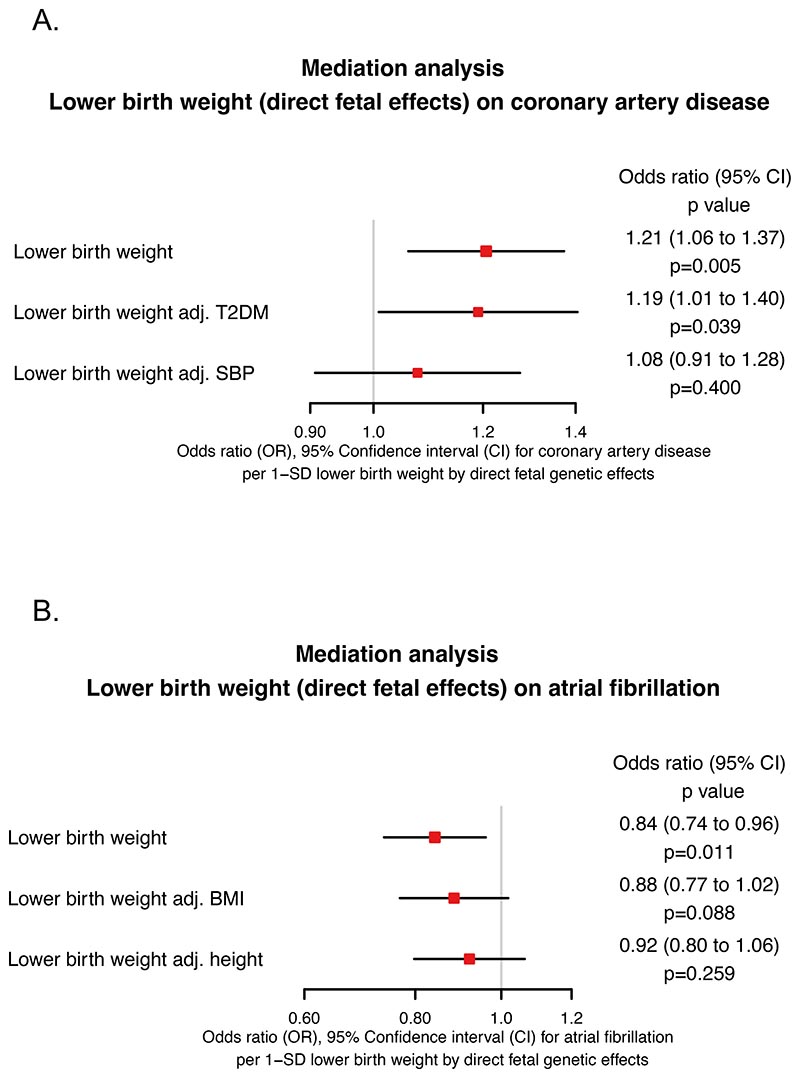
Mendelian randomization estimates for the effects of (A) genetically-predicted birth weight and (B) genetically-predicted birth weight after isolating only variants that act through direct fetal effects, on cardiovascular magnetic resonance imaging parameters of cardiac structure and function. LA Max = left atrial maximum volume, LATEF = left atrial total ejection fraction, LVESV = left ventricular end systolic volume, LVEDV = left ventricular end diastolic volume, LVSV = left ventricular stroke volume, LVEF = left ventricular ejection fraction, LV Mass = left ventricular mass, RA Max = right atrial maximum area, RA Min = right atrial minimum area, RA FAC = right atrial fractional area change, RVESV = right ventricular end systolic volume, RVEDV = right ventricular end diastolic volume, RVSV = right ventricular systolic volume, RVEF = right ventricular ejection fraction, Prox PA Diam = proximal pulmonary artery diameter, Asc Aorta Diam = ascending aorta diameter, SD = standard deviation.

Though there were statistically significant associations with other measures of cardiac function, the associations between birth weight and LA Max, LATEF, LVEF, proximal PA diameter and ascending aorta diameter were not statistically significant. The results are reported in Figure 3A.

When isolating the direct fetal genetic effects, only the association of lower birth weight with lower RA Max (β −0.17 [−0.28 to −0.07] p=4.71×10^−3^), lower RA Min (β −0.24 [−0.33 to −0.14] p=1.35×10^−5^), and higher RA FAC (β 0.21 [0.11 to 0.30] p=1.05×10^−4^) remained consistent with statistical significance despite the loss of power when accounting for multiple testing. The association with LV mass was reversed in direction (β −0.14 [−0.29 to 0.01] p=0.101) although this failed to reach statistical significance and crossed the null. Associations with other variables remained consistent in direction and magnitude, but were no longer statistically significant, however this is expected given the loss of power when accounting for multiple testing. The results are reported in Figure 3B.

### Replication and sensitivity analyses

The replication analysis using gene-exposure data from Juliusdottir *et al.*’s study produced consistent findings, except for the outcomes of ischaemic stroke and LV Mass as shown in Supplementary Figures 2 and 3. While the association between genetically-predicted birth weight and stroke was not significant on primary analysis, in the replication analysis lower genetically-predicted birth weight was associated with a greater risk of stroke (OR 1.17 [1.05 to 1.31] p=0.018^2^). This association was not significant after isolating direct fetal effects only (OR 1.06 [0.90 to 1.24] p=0.580), consistent with the primary analysis. The findings for LV mass were consistent with the direction of the primary analysis: lower genetically predicted birth weight overall was associated with a higher LV Mass (β 0.13 [0.07 to 0.20] p=2.55×10^−4^) but lower genetically-predicted birth weight through direct fetal effects was also associated with lower LV mass (β −0.13 [−0.23 to −0.03] p=0.021). Notably these results reached statistical significance in the replication analysis, despite only a suggestion of this reversal of effect in the main analysis where the direct fetal effects on LV mass followed a similar reversing pattern but failed to reach statistical significance.

The sensitivity analyses using weighted median MR and MR-Egger produced consistent estimates, and MR-Egger intercept test did not identify evidence suggestive of directional pleiotropy, as reported in Supplementary Table 2 for the main analyses, and Supplementary Table 3 for the replication analyses.

In the main analysis, the mean instrument F-statistic was 58 for birth weight overall, and 142 for birth weight after isolating direct fetal effects only. In the replication analysis, the instrument F-statistic for genetically-predicted birth weight was 91, and after isolating direct fetal effects only this was 100.

### Mediation analysis

**Figure 4:**
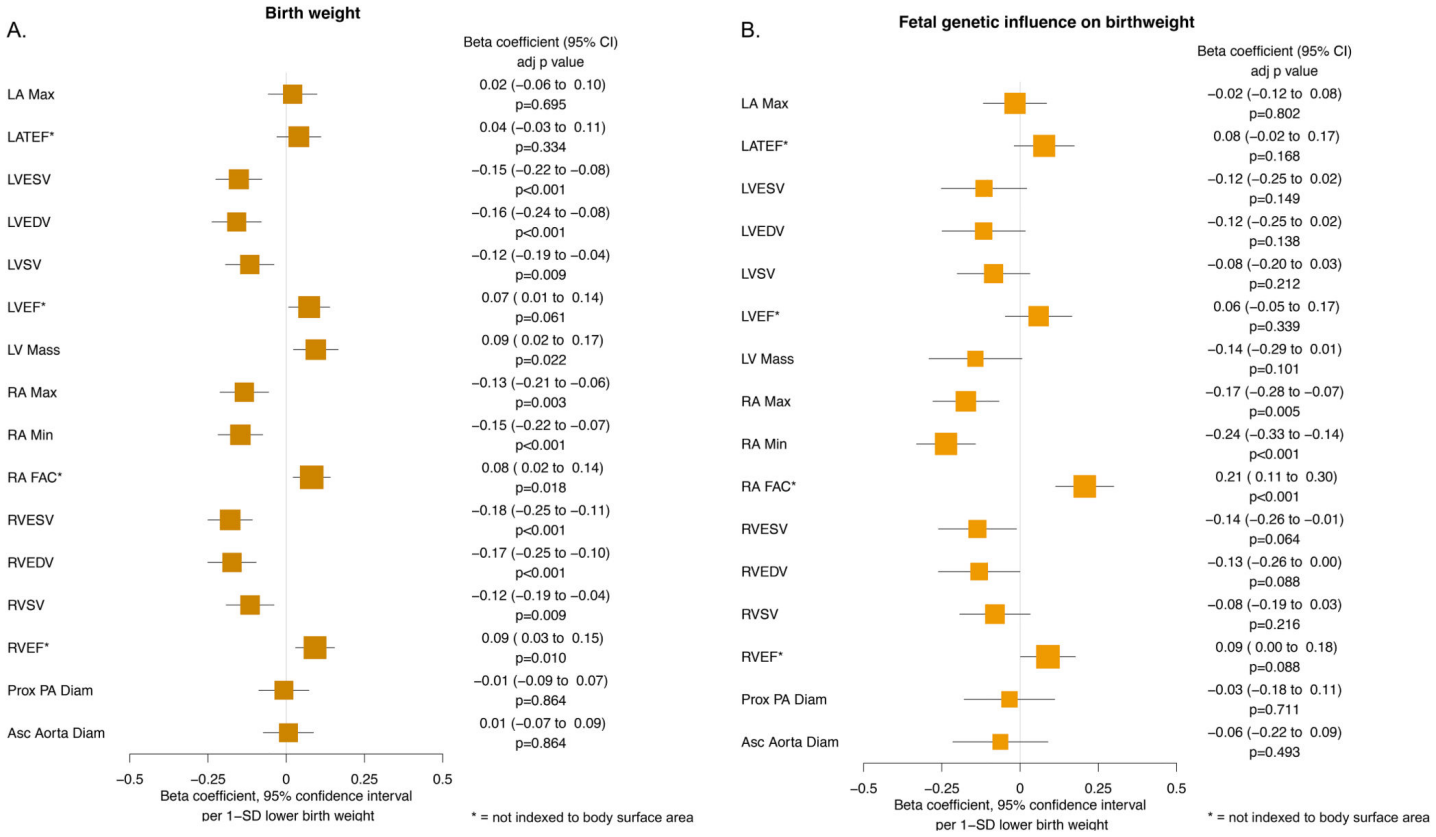
Mediation analysis utilising multivariable mendelian randomization, displaying estimates for the effects of genetically-predicted birth weight after isolating only direct fetal effects, on (1) Coronary artery disease and (2) Atrial fibrillation, before and after accounting for putative mediating factors. BMI = Body mass index, T2DM = type 2 diabetes, SBP = systolic blood pressure, SD = standard deviation.

For the association of lower birth weight and higher risk of coronary artery disease, potential mediation was explored through SBP and T2DM, as these factors are known to be inversely associated with birth weight^14^, but directly associated with coronary artery disease, and thus provide feasible pathways for mediation of an overall inverse association. On mediation analysis, the association between birth weight through direct fetal effects and coronary artery disease (unadjusted OR 1.21 [1.06 to 1.37] p=0.005) was mildly attenuated after adjustment for T2DM (adjusted OR 1.19 [1.01 to 1.40] p=0.039; conditional F-statistic for birth weight = 36.9), and to a greater degree after adjustment for SBP (adjusted OR 1.08 [0.91 to 1.28] p=0.400; conditional F-statistic for birth weight = 46.1). This suggests that partial mediation might exist by both of the considered risk factors. The results are reported in Figure 4A.

For the association between lower birth weight and lower risk of AF, potential mediation was explored through BMI and height, as these factors are both directly associated with birth weight^14^, and are also directly associated with higher risk of AF, and thus provide feasible pathways for mediation of an overall direct association. On mediation analysis, the association between birth weight through direct fetal effects and atrial fibrillation (unadjusted OR 0.84 [0.74 to 0.96] p=0.011) was mildly attenuated after adjustment for BMI (adjusted OR 0.88 [0.77 to 1.02] p=0.088; conditional F-statistic for birth weight = 53.5), and to a greater degree after adjusting for height (adjusted OR 0.92 [0.80 to 1.06] p=0.259; conditional F-statistic for birth weight = 13.2). This suggests that partial mediation might exist by both of the considered risk factors. The results are reported in Figure 4B.

## Discussion

The purpose of this study was to further understand the mechanistic pathways underlying the association of birth weight with cardiovascular disease, with the specific aim of establishing whether birth weight had a direct, causal role on determining cardiovascular risk that is independent of the intrauterine environment. There are several key messages. First, the results suggest that birth weight has direct causal effects on cardiovascular disease that are independent of intrauterine exposures. This provides important insight regarding the mechanisms underlying the DOHaD hypothesis. Though this result does not negate a potential role of the intrauterine environment, which might be a risk enhancer, or act to modify cardiovascular risk through independent pathways, they indicate that birth weight exerts at least some effects that are independent of the intrauterine environment. Second, we establish that the association of these direct fetal effects with coronary artery disease may be partly mediated by T2DM and SBP, identifying these as key targets for surveillance and targeted prevention. Third, we identify an association of higher birth weight with greater risk of atrial fibrillation, mediated by BMI and, to a greater extent, height. Finally, we provide evidence that low birth weight contributes to adverse cardiac remodelling, broadly following a pattern of smaller chamber volumes, lower stroke volumes, and greater contractility.

There are two major strengths. The first of these is its genetic epidemiological approach, which distinguishes it from the vast literature of prior observational studies exploring the cardiometabolic consequences of low birth weight. The use of MR in this setting mitigates the potential impact of confounding, which is an important concern when studying birth weight because there are many potential difficult-to-measure confounders that may influence the association between birth weight and cardiovascular disease. In the hierarchy of evidence, MR has been advocated as providing “critical” evidence on risk factor–outcome relationships^7^, especially when, as in this case, the risk factor in question is not practically or ethically amenable to randomisation. The second strength is the specific investigation of the direct effects of birth weight: this provides crucial mechanistic insight for the DOHaD hypothesis that has implications for risk stratification. Though it does not imply that intrauterine exposures have no influence, it does suggest that individuals with low birth weight are at enhanced risk whether their low birth weight is related to an adverse intrauterine environment or not. Finally, the investigations of potential modifiable mediating pathways adds clinical relevance by identifying a number of key mediators that are amenable to intervention.

Lower genetically-predicted birth weight was associated with greater coronary artery disease risk. Conversely, higher genetically-predicted birth weight was associated with greater atrial fibrillation risk. These results are in line with observational evidence^2–6,39–44^, as well as previous evidence from MR studies^10,13^. Our study specifically adds by establishing a contribution of direct causal effects on birth weight. The association of lower birth weight with higher coronary artery disease risk is in line with a previous investigation of 26,057 mother–offspring pairs from the Nord-Trøndelag Health (HUNT) Study, where Moen *et al*.^15^ demonstrated that offspring genetic risk score was independently associated with offspring cardiometabolic factors, including glycaemic and cholesterol traits, in models adjusting for maternal and paternal genetic risk scores. The pattern of these results mirrors ours, and we extend this work by revealing consistent findings for both disease outcomes and imaging markers of adverse cardiac structure and function.

Though the association of lower birth weight with lower risk of atrial fibrillation might not seem intuitively consistent with the result for coronary artery disease, it is directionally consistent with previous observational^41,42^ and MR studies^13^. From a mechanistic perspective, it is also consistent with the known role of body anthropometric measures in determining risk of atrial fibrillation. It is indeed well recognised that greater body weight and height are strongly associated with atrial fibrillation risk^45–47^. The important role of BMI and height in driving this association was highlighted in our mediation analysis, where we observed an attenuation in the magnitude of the association estimate after conditioning on BMI and, to a greater extent, height.

The association between low birth weight and cardiac structure and function has been studied in the observational setting. Recently, Raisi-Estabragh *et al.* reported that in a cohort of 19,238 participants in the UK Biobank, lower birth weight was associated with more concentric pattern of LV remodelling (higher LVM/LVEDV) and poorer LV function (lower LVSV index)^44^. Prior to this, the majority of available evidence remained restricted to preterm birth or small-for-gestational-age (SGA) individuals and preterm birth. In a study by Arnott *et al.*, SGA adults had larger LV volumes and lower LVSV compared to individuals of average size for gestational age^48^. Lewandowski *et al.* reported a greater LV mass, smaller chamber volumes and worse LV strain in individuals with a history of preterm birth compared with controls^49^, findings that were replicated in relation to the atria^50^, in a subsequent study in an adolescent^51^ and a further adult cohort^52^. Importantly, these changes are not benign: they have been shown to relate to a reduced myocardial functional reserve and an increase in diffuse myocardial fibrosis^53,54^. However, we did not identify a significant association with heart failure. This might suggest that the adverse remodelling changes are subclinical in nature, but might also relate to insufficient power to detect an association. In our study, we additionally noted that with lower birth weight, despite smaller chamber volumes and stroke volumes, there was an association with metrics of greater contractility. Interestingly, prior observational work has identified similar patterns for preterm born adults^55^, where despite smaller RA volumes, RA reservoir and booster strain were higher in the preterm cohort, suggesting a degree of functional compensation for the smaller RA volumes. Prior observational evidence suggests that preterm-born adults, when exposed to physiologic stress, may exhibit a response of exaggerated contraction, possibly indicating compensation for reductions in volumetric reserve^50,55^.

In the replication analysis, we noted that lower genetically-predicted birth weight overall was associated with greater LV mass. When isolating direct fetal genetic effects this association was fully reversed, with lower genetically-predicted birth weight associating with lower LV mass. This reversal was present in the main analysis, with consistent magnitude and direction, though when isolating for direct fetal genetic effects the association did not reach statistical significance. This reversal pattern is an intriguing finding, which we suggest it might relate to differential vertically pleiotropic signals through SBP. The ‘overall’ genetically-predicted birth weight instruments include indirect maternal effects. Previous work conducted by Warrington *et al.*^14^ identified a strong generational effect relating to SBP and maternal genetic influences on birth weight: high maternal SBP was causally associated with lower offspring birth weight, and subsequent transmission of maternally-inherited SBP variants to the offspring begets higher offspring SBP in later life. Thus, when looking at birth weight overall, the maternally-inherited SBP genetic risk might feasibly drive the association with higher LV mass through the vertically pleiotropic phenotype of SBP. Subsequently, when isolating direct fetal genetic effects, we remove the influence of maternally-inherited SBP associated alleles, and therefore this influence is lost. Now, we expect predominantly anthropometric traits to drive the association. Thus, we observe a direct association, with lower genetically-predicted birth weight associated with lower LV mass (and conversely, high genetically-predicted birth weight associates with higher LV mass). Unfortunately, due to the lack of availability of individual-level data to isolate maternal genetic effects in the present study, this hypothesis could not be formally tested within the scope of our work. We therefore highlight this as a key research priority for individual-level studies.

There are some limitations to consider. First, in order to limit potential influence from population stratification, the data sources for our analysis were restricted to populations of European ancestry. Though this limits potential bias from population stratification, it means that the results may not be generalisable to populations of other ancestries. Second, the confidence with which causal relationships can be drawn from MR results depends on the plausibility of the instrumental variable assumptions^56^. We explored the first of these assumptions through checking instrument strength using F-statistics^23^, and the third of these through the use of multiple sensitivity analyses more robust to pleiotropy. In these analyses, we did not identify issues relating to weak instruments of directional pleiotropy and we therefore do not expect violation of this assumption. Third, the lack of individual-level data for the analyses is a limitation as it precludes formal quantification of the role of maternal effects on birth weight (and thus the intrauterine environment) as well as modelling of interactions between maternal and fetal genomes, or potentially non-linear effects. Fourth, because of the nature of the cardiovascular outcomes in the original GWASs, we could not formally quantify proportions mediated in the mediation analyses, due to the issue of non-collapsibility of odds ratios in the setting of binary, non-rare outcomes. Fifth, when performing mediation analyses, it is important to note that attenuation of effects observed after adjustment for the potential mediator might not stem from true mediation (vertical pleiotropy) but rather from horizontal pleiotropy. The lack of evidence of horizontal pleiotropy in our sensitivity analyses supports a mediating, rather than horizontally pleiotropic, role of SBP and the other phenotypes explored in the mediation analyses. Sixth, it is important to note that the magnitude of odds ratios represent lifetime associations that are not specific to particular age brackets or a defined time span. Thus, future studies are needed to investigate how the timing of cardiovascular risk might differ in individuals with low birth weight. Finally, due to the original design of the data sources used, there can be some ‘healthy participant’ bias, with the study volunteers being a higher socioeconomic status and healthier compared to the general population. This could lead to some bias in the effect size estimates.

From a clinical perspective, our study suggests that individuals born with low birth weight are at enhanced cardiovascular risk, independent of whether their low birth weight relates to intrauterine pathology. This encourages further consideration of birth weight in risk stratification for cardiovascular disease. It has been previously suggested that preterm-born individuals should be under more intensive follow up for early blood pressure control and routine surveillance of cardiovascular structure and function using echocardiography and cardiopulmonary exercise testing (CPET)^57^. However, no guidelines currently use birth weight itself as a risk-enhancing factor; but observational - and now genetic - evidence suggests that this at-risk population might benefit from early targeted risk stratification and more aggressive prevention strategies. Within our study, we demonstrate that at least a part of the associations are mediated by modifiable risk factors including SBP and T2DM. This encourages early intervention on these factors in low birth weight individuals, as this is likely to at least partly mitigate the enhanced cardiovascular risk related to low birth weight.

In our study, we utilized MR to investigate the relationship between birth weight and cardiovascular disease, and we describe several direct, causal associations between birth weight and cardiovascular disease that are independent of the intrauterine environment. We also investigate the relationship between birth weight and 16 measurements of cardiac structure and function. Our findings suggest that lower birth weight plays a direct, causal role in the development of coronary artery disease, but that conversely higher birth weight is associated with atrial fibrillation. These direct associations provide insight into them mechanisms underlying the DOHaD hypothesis, suggesting that low birth weight is causally related to cardiovascular disease risk and cardiac structure and function, even after isolating out the potential effects of the intrauterine environment. Importantly, we also identify a number of modifiable cardiometabolic mediators that provide important targets for early intervention in the clinical setting.

## Supplementary Material

Supplementary figure

Supplementary figure 1

Supplementary figure 2

Supplementary figure 3

Supplementary table 1

Supplementary table 2

## Figures and Tables

**Table 1:** Information on the studies and consortia from which genetic association data were obtained. EUR = European.

Phenotype	Study or consortium	Ancestry	Cases / controls	Case definition	Control definition	Units	Link / PMID
**Exposures**							
**Birth Weight**	Early Growth Genetics and UK Biobank^13^	EUR	321,223	Own birth weight	n/a	1-SD	31043758
**Birth Weight: fetal effects only**	Early Growth Genetics and UK Biobank^13^	EUR	321,223	Own birth weight, after adjustment for maternal genetic effects using structural equation modelling	n/a	1-SD	31043758
**Exposures for replication analysis**							
**Birth Weight**	Icelandic birth register, Early Growth Genetics and UK Biobank^15^	EUR	423,683	Own birth weight	n/a	1-SD	34282336
**Birth Weight: fetal effects only**	Icelandic birth register, Early Growth Genetics and UK Biobank^15^	EUR	104,920	Own birth weight; selecting variants that only influence birth weight via fetal genome (clusters 1,2 and 3)	n/a	1-SD	34282336
**Outcomes**							
**Atrial fibrillation**	Nielsen *et al.^23^*	EUR	60,620 / 970,216	Clinically diagnosed atrial fibrillation or flutter UKB and HUNT cohorts: ICD-9 427.3 ICD-10 I48	No history of atrial fibrillation, flutter or other arrhythmias	Log(OR)	30061737
**Coronary artery disease**	Van der Harst *et al.^24^*	EUR	122,733 / 424,528	Coronary artery disease or myocardial infarction	No known coronary artery disease or past myocardial infarction	Log(OR)	29212778
**Heart failure**	Levin *et al.^25^*	EUR	115,150 / 1,550,331	Diagnosis of heart failure by physician, or healthcare record, and corroborated on self-report	No history of heart failure	Log(OR)	36376295
**Ischaemic stroke**	Malik *et al.^26^*	EUR	34,217 / 406,111	Any ischaemic stroke	No history of stroke, of any type	Log(OR)	29531354
**Cardiac structure and function**	Pirruccello *et al.*	EUR	45,504	UK Biobank participants	n/a	Log(OR)	35697867
**Left atrial maximum volume and left atrial total ejection fraction**	Ahlberg *et al.^2^*	EUR	35,658	UK Biobank participants	n/a	Log(OR)	34338756
**Left ventricular mass**	Khurshid *et al.^29^*	EUR	43,230	UK Biobank participants	n/a	Log(OR)	36944631
**Mediators**							
**Body mass index**	Pulit *et al.^22^*	EUR	434,794	n/a	n/a	1-SD	30239722
**Height**	Yengo *et al.^33^*	EUR	709,594	n/a	n/a	1-SD	30124842
**Systolic blood pressure**	Evangelou *et al.^34^*	EUR	757,601	n/a	n/a	1-mmHg	30224653
**Type 2 diabetes**	Mahajan *et al.^35^*	EUR	80,154 / 853,816	Type 2 diabetes	No history of type 2 diabetes	Log(OR)	35551307
