# Supplementary material for: Birth weight influences cardiac structure, function and disease risk: evidence of a causal association
Source: Eur Heart J. Author manuscript; Available in PMC 2024 Feb 21. (PMC10849320; doi:10.1093/eurheartj/ehad631)
Supplement: Supplementary figure [file EMS190943-supplement-Supplementary_figure.docx]

Supplementary Figure 1 – Replication analysis: Diagram representing study design and data analysis workflow. EGG = Early Growth Genetics, UKB = UK Biobank, SNP = single nucleotide polymorphism.

Supplementary Figure 2 – Replication analysis: Mendelian randomization estimates for the effects of (A) genetically-predicted birth weight and (B) genetically-predicted birth weight after isolating direct fetal effects, on cardiovascular outcomes. SD = standard deviation.


Supplementary Figure 3 - Replication analysis: Mendelian randomization estimates for the effects of (A) genetically-predicted birth weight and (B) genetically-predicted birth weight after isolating direct fetal effects, on cardiovascular magnetic resonance imaging parameters of cardiac structure and function. LA Max = left atrial maximum volume, LATEF = left atrial total ejection fraction, LVESV = left ventricular end systolic volume, LVEDV = left ventricular end diastolic volume, LVSV = left ventricular stroke volume, LVEF = left ventricular ejection fraction, LV Mass = left ventricular mass, RA Max = right atrial maximum area, RA Min = right atrial minimum area, RA FAC = right atrial fractional area change, RVESV = right ventricular end systolic volume, RVEDV = right ventricular end diastolic volume, RVSV = right ventricular systolic volume, RVEF = right ventricular ejection fraction, Prox PA Diam = proximal pulmonary artery diameter, Asc Aorta Diam = ascending aorta diameter, SD = standard deviation
