# Supplementary material for: Birth weight influences cardiac structure, function and disease risk: evidence of a causal association
Source: Eur Heart J. Author manuscript; Available in PMC 2024 Feb 21. (PMC10849320; doi:10.1093/eurheartj/ehad631)
Supplement: Supplementary figure 1 [file EMS190943-supplement-Supplementary_figure_1.pdf]

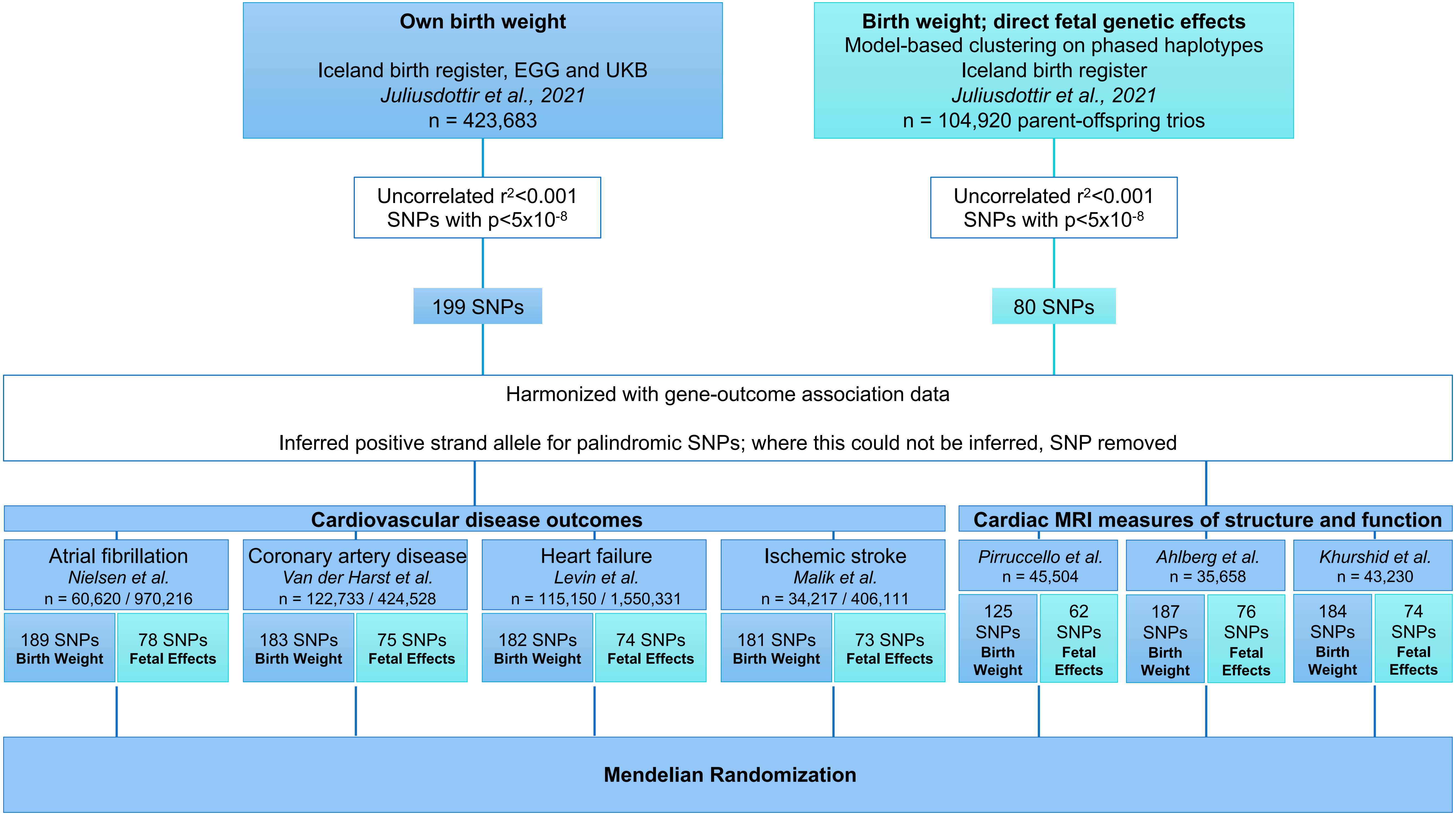
Own birth weight
Iceland birth register, EGG and UKB
Juliusdottir et al., 2021
n = 423,683
Birth weight; direct fetal genetic effects
Model-based clustering on phased haplotypes
Iceland birth register
Juliusdottir et al., 2021
n = 104,920 parent-offspring trios
Uncorrelated r2<0.001
SNPs with p<5x10-8
Uncorrelated r2<0.001
SNPs with p<5x10-8
199 SNPs
80 SNPs
Harmonized with gene-outcome association data
Inferred positive strand allele for palindromic SNPs; where this could not be inferred, SNP removed
Cardiovascular disease outcomes
Cardiac MRI measures of structure and function
Atrial fibrillation
Nielsen et al.
n = 60,620 / 970,216
Coronary artery disease
Van der Harst et al.
n = 122,733 / 424,528
Heart failure
Levin et al.
n = 115,150 / 1,550,331
Ischemic stroke
Malik et al.
n = 34,217 / 406,111
Pirruccello et al.
n = 45,504
Ahlberg et al.
n = 35,658
Khurshid et al.
n = 43,230
189 SNPs Birth Weight
78 SNPs Fetal Effects
183 SNPs Birth Weight
75 SNPs Fetal Effects
182 SNPs Birth Weight
74 SNPs Fetal Effects
181 SNPs Birth Weight
73 SNPs Fetal Effects
125 SNPs Birth Weight
62 SNPs Fetal Effects
187 SNPs Birth Weight
76 SNPs Fetal Effects
184 SNPs Birth Weight
74 SNPs Fetal Effects
Mendelian Randomization
