# Supplementary figures and images for: Birth weight influences cardiac structure, function and disease risk: evidence of a causal association
Source: Eur Heart J. Author manuscript; Available in PMC 2024 Feb 21. (PMC10849320; doi:10.1093/eurheartj/ehad631)

A.

## Birth weight

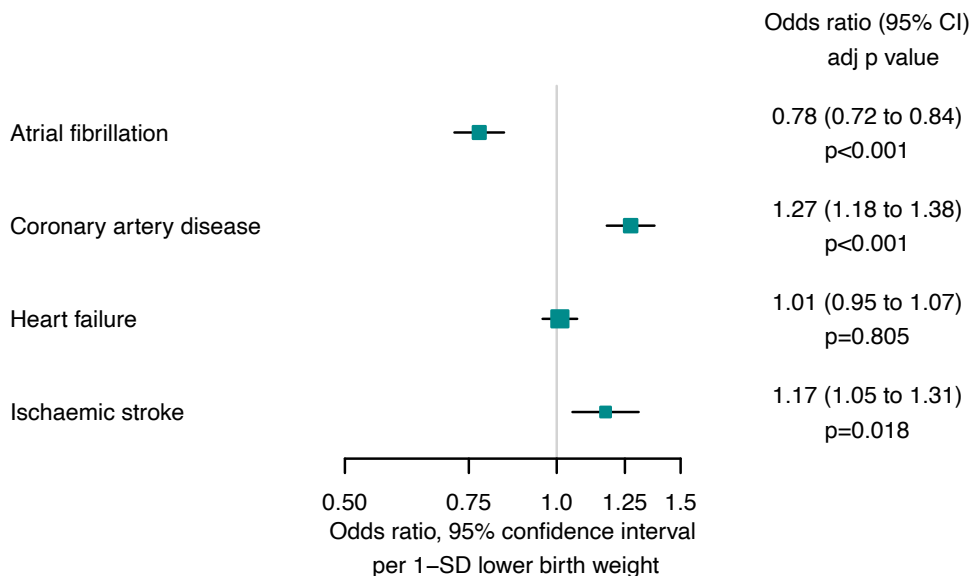

B.

## Fetal genetic influence on birth weight

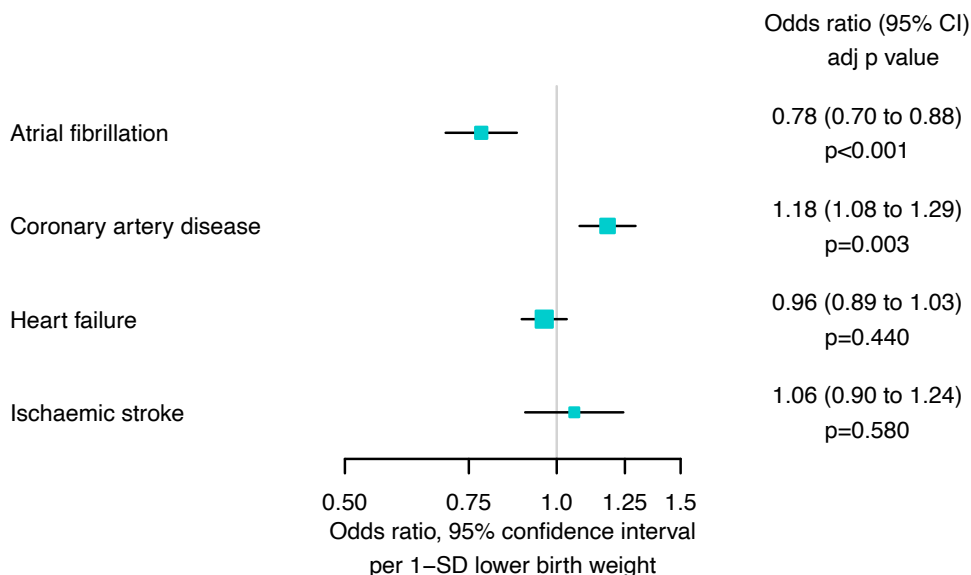

Supplement: Supplementary figure 2 [file EMS190943-supplement-Supplementary_figure_2.pdf]

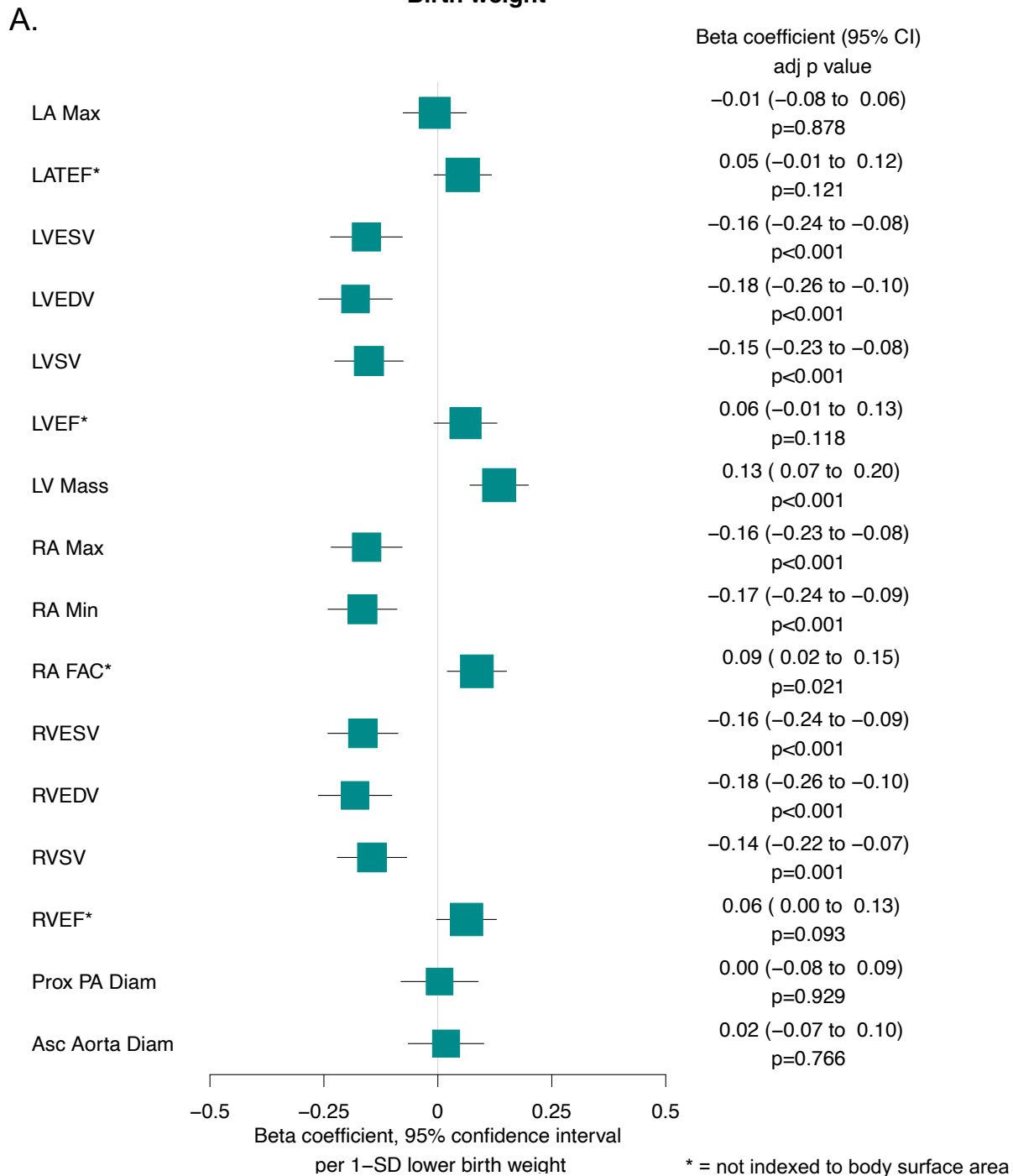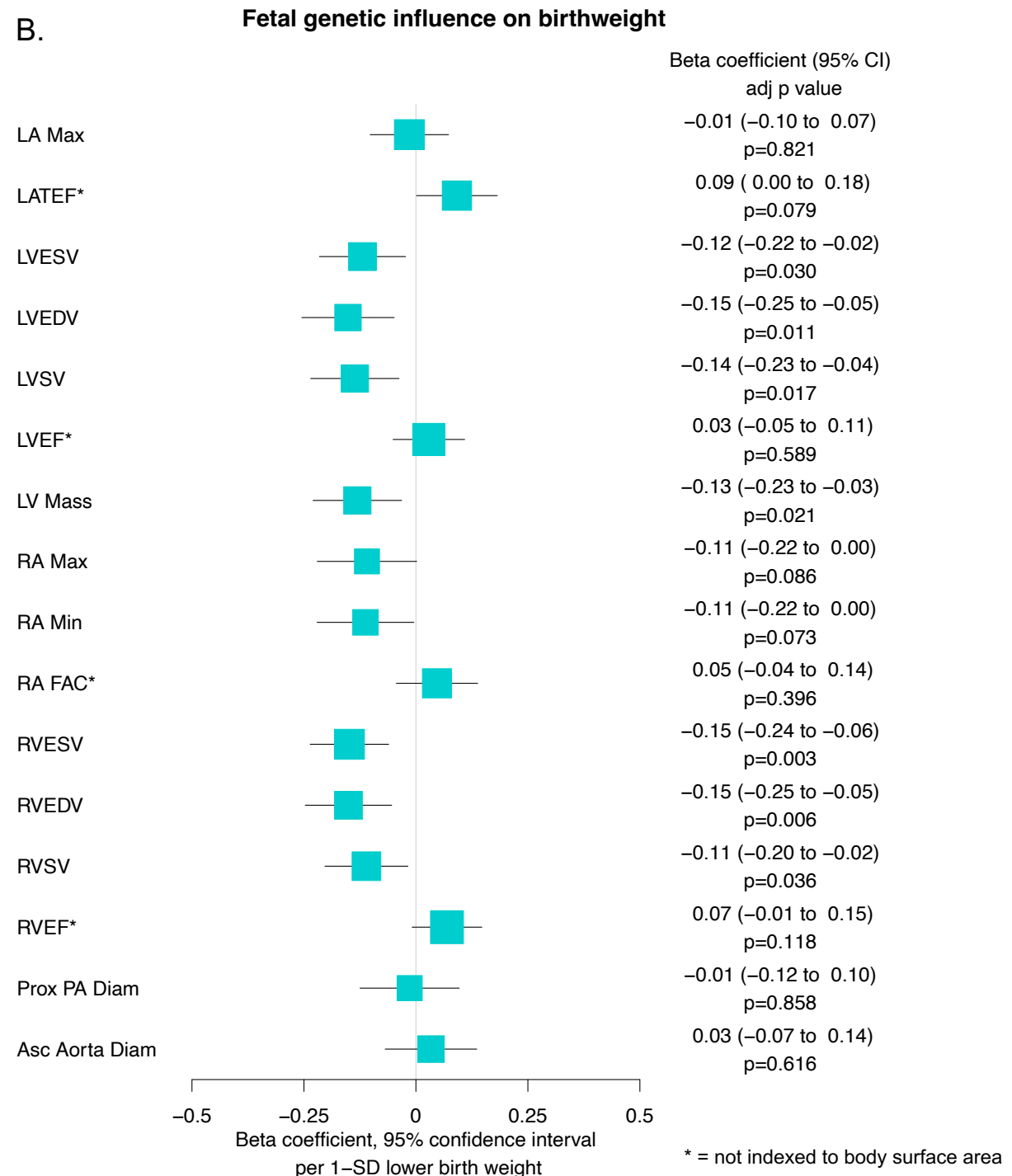

Supplement: Supplementary figure 3 [file EMS190943-supplement-Supplementary_figure_3.pdf]
