# Supplementary material for: Birth weight influences cardiac structure, function and disease risk: evidence of a causal association
Source: Eur Heart J. Author manuscript; Available in PMC 2024 Feb 21. (PMC10849320; doi:10.1093/eurheartj/ehad631)
Supplement: Supplementary table 1 [file EMS190943-supplement-Supplementary_table_1.docx]

Supplementary Table 1 – Mendelian randomization sensitivity analyses used to assess for potential presence of directional pleiotropy in the main analysis, using Weighted median and MR Egger

| **Exposure** | **Outcome** | **Method** | **B coefficient** | **Std Err** | **pval** |
| --- | --- | --- | --- | --- | --- |
| Birth weight | Atrial fibrillation | Weighted median | -0.245 | 0.050 | 7.39E-07 |
|  |  | MR Egger | -0.447 | 0.134 | 0.001 |
|  |  | intercept | 0.005 | 0.003 | 0.092 |
|  | Coronary artery disease | Weighted median | 0.165 | 0.042 | 9.74E-05 |
|  |  | MR Egger | 0.062 | 0.130 | 0.633 |
|  |  | intercept | 0.005 | 0.003 | 0.125 |
|  | Heart failure | Weighted median | 0.029 | 0.037 | 0.430 |
|  |  | MR Egger | -0.148 | 0.097 | 0.130 |
|  |  | intercept | 0.005 | 0.002 | 0.040 |
|  | Ischaemic stroke | Weighted median | 0.134 | 0.071 | 0.060 |
|  |  | MR Egger | 0.036 | 0.192 | 0.852 |
|  |  | intercept | 0.002 | 0.004 | 0.680 |
|  | LA Max Indexed | Weighted median | 0.020 | 0.049 | 0.686 |
|  |  | MR Egger | 0.010 | 0.108 | 0.923 |
|  |  | intercept | 0.000 | 0.002 | 0.921 |
|  | LATEF | Weighted median | 0.052 | 0.051 | 0.314 |
|  |  | MR Egger | 0.060 | 0.097 | 0.539 |
|  |  | intercept | 0.000 | 0.002 | 0.830 |
|  | LVESV Indexed | Weighted median | -0.113 | 0.046 | 0.014 |
|  |  | MR Egger | -0.108 | 0.102 | 0.289 |
|  |  | intercept | -0.001 | 0.002 | 0.648 |
|  | LVEDV Indexed | Weighted median | -0.114 | 0.046 | 0.014 |
|  |  | MR Egger | -0.118 | 0.109 | 0.279 |
|  |  | intercept | -0.001 | 0.002 | 0.690 |
|  | LVSV Indexed | Weighted median | -0.133 | 0.048 | 0.005 |
|  |  | MR Egger | -0.115 | 0.107 | 0.286 |
|  |  | intercept | 0.000 | 0.002 | 0.984 |
|  | LVEF | Weighted median | 0.052 | 0.047 | 0.263 |
|  |  | MR Egger | 0.042 | 0.091 | 0.643 |
|  |  | intercept | 0.001 | 0.002 | 0.711 |
|  | LV Mass Indexed | Weighted median | 0.066 | 0.044 | 0.138 |
|  |  | MR Egger | 0.202 | 0.108 | 0.064 |
|  |  | intercept | -0.003 | 0.002 | 0.291 |
|  | RA Max Indexed | Weighted median | -0.087 | 0.048 | 0.072 |
|  |  | MR Egger | -0.279 | 0.106 | 0.009 |
|  |  | intercept | 0.004 | 0.002 | 0.143 |
|  | RA Min Indexed | Weighted median | -0.146 | 0.050 | 0.003 |
|  |  | MR Egger | -0.344 | 0.097 | 0.001 |
|  |  | intercept | 0.005 | 0.002 | 0.030 |
|  | RA FAC | Weighted median | 0.123 | 0.047 | 0.009 |
|  |  | MR Egger | 0.231 | 0.082 | 0.005 |
|  |  | intercept | -0.004 | 0.002 | 0.050 |
|  | RVESV Indexed | Weighted median | -0.170 | 0.043 | 0.000 |
|  |  | MR Egger | -0.216 | 0.098 | 0.029 |
|  |  | intercept | 0.001 | 0.002 | 0.686 |
|  | RVEDV Indexed | Weighted median | -0.121 | 0.045 | 0.007 |
|  |  | MR Egger | -0.168 | 0.107 | 0.117 |
|  |  | intercept | 0.000 | 0.002 | 0.957 |
|  | RVSV Indexed | Weighted median | -0.101 | 0.048 | 0.034 |
|  |  | MR Egger | -0.059 | 0.105 | 0.578 |
|  |  | intercept | -0.001 | 0.002 | 0.562 |
|  | RVEF | Weighted median | 0.083 | 0.047 | 0.080 |
|  |  | MR Egger | 0.168 | 0.086 | 0.051 |
|  |  | intercept | -0.002 | 0.002 | 0.340 |
|  | Prox PA Diam Indexed | Weighted median | -0.027 | 0.049 | 0.580 |
|  |  | MR Egger | -0.149 | 0.110 | 0.176 |
|  |  | intercept | 0.003 | 0.003 | 0.341 |
|  | Asc Aorta Diam Indexed | Weighted median | -0.032 | 0.049 | 0.519 |
|  |  | MR Egger | 0.009 | 0.110 | 0.938 |
|  |  | intercept | 0.000 | 0.003 | 0.988 |
| Fetal genetic influence on birth weight | Atrial fibrillation | Weighted median | -0.228 | 0.069 | 0.001 |
|  |  | MR Egger | -0.250 | 0.202 | 0.229 |
|  |  | intercept | 0.003 | 0.007 | 0.686 |
|  | Coronary artery disease | Weighted median | 0.109 | 0.061 | 0.072 |
|  |  | MR Egger | 0.119 | 0.188 | 0.533 |
|  |  | intercept | 0.003 | 0.007 | 0.700 |
|  | Heart failure | Weighted median | 0.014 | 0.054 | 0.797 |
|  |  | MR Egger | -0.024 | 0.157 | 0.878 |
|  |  | intercept | 0.001 | 0.005 | 0.831 |
|  | Ischaemic stroke | Weighted median | -0.007 | 0.114 | 0.952 |
|  |  | MR Egger | 0.110 | 0.395 | 0.784 |
|  |  | intercept | -0.006 | 0.014 | 0.683 |
|  | LA Max Indexed | Weighted median | 0.014 | 0.070 | 0.841 |
|  |  | MR Egger | -0.040 | 0.141 | 0.776 |
|  |  | intercept | 0.001 | 0.005 | 0.855 |
|  | LATEF | Weighted median | 0.064 | 0.067 | 0.344 |
|  |  | MR Egger | 0.028 | 0.131 | 0.830 |
|  |  | intercept | 0.002 | 0.005 | 0.693 |
|  | LVESV Indexed | Weighted median | -0.088 | 0.072 | 0.224 |
|  |  | MR Egger | 0.354 | 0.160 | 0.037 |
|  |  | intercept | -0.018 | 0.006 | 0.004 |
|  | LVEDV Indexed | Weighted median | -0.177 | 0.075 | 0.019 |
|  |  | MR Egger | 0.313 | 0.159 | 0.062 |
|  |  | intercept | -0.017 | 0.006 | 0.008 |
|  | LVSV Indexed | Weighted median | -0.010 | 0.071 | 0.884 |
|  |  | MR Egger | 0.143 | 0.154 | 0.362 |
|  |  | intercept | -0.009 | 0.006 | 0.125 |
|  | LVEF | Weighted median | 0.060 | 0.069 | 0.383 |
|  |  | MR Egger | -0.234 | 0.134 | 0.094 |
|  |  | intercept | 0.011 | 0.005 | 0.027 |
|  | LV Mass Indexed | Weighted median | -0.063 | 0.073 | 0.391 |
|  |  | MR Egger | 0.293 | 0.256 | 0.265 |
|  |  | intercept | -0.016 | 0.009 | 0.090 |
|  | RA Max Indexed | Weighted median | -0.136 | 0.073 | 0.065 |
|  |  | MR Egger | -0.006 | 0.143 | 0.967 |
|  |  | intercept | -0.007 | 0.005 | 0.219 |
|  | RA Min Indexed | Weighted median | -0.207 | 0.072 | 0.004 |
|  |  | MR Egger | -0.027 | 0.128 | 0.832 |
|  |  | intercept | -0.008 | 0.005 | 0.091 |
|  | RA FAC | Weighted median | 0.152 | 0.068 | 0.027 |
|  |  | MR Egger | 0.040 | 0.127 | 0.759 |
|  |  | intercept | 0.007 | 0.005 | 0.171 |
|  | RVESV Indexed | Weighted median | -0.066 | 0.070 | 0.344 |
|  |  | MR Egger | 0.206 | 0.157 | 0.202 |
|  |  | intercept | -0.013 | 0.006 | 0.028 |
|  | RVEDV Indexed | Weighted median | -0.126 | 0.075 | 0.095 |
|  |  | MR Egger | 0.237 | 0.163 | 0.159 |
|  |  | intercept | -0.014 | 0.006 | 0.023 |
|  | RVSV Indexed | Weighted median | -0.079 | 0.073 | 0.280 |
|  |  | MR Egger | 0.187 | 0.146 | 0.212 |
|  |  | intercept | -0.010 | 0.005 | 0.060 |
|  | RVEF | Weighted median | 0.073 | 0.065 | 0.256 |
|  |  | MR Egger | -0.097 | 0.120 | 0.426 |
|  |  | intercept | 0.007 | 0.004 | 0.109 |
|  | Prox PA Diam Indexed | Weighted median | 0.007 | 0.074 | 0.920 |
|  |  | MR Egger | 0.133 | 0.200 | 0.512 |
|  |  | intercept | -0.006 | 0.007 | 0.378 |
|  | Asc Aorta Diam Indexed | Weighted median | -0.051 | 0.067 | 0.445 |
|  |  | MR Egger | 0.042 | 0.213 | 0.846 |
|  |  | intercept | -0.004 | 0.008 | 0.601 |
