# Supplementary material for: Birth weight influences cardiac structure, function and disease risk: evidence of a causal association
Source: Eur Heart J. Author manuscript; Available in PMC 2024 Feb 21. (PMC10849320; doi:10.1093/eurheartj/ehad631)
Supplement: Supplementary table 2 [file EMS190943-supplement-Supplementary_table_2.docx]

Supplementary Table 2 – Mendelian randomization sensitivity analyses used to assess for potential presence of directional pleiotropy in the replication analysis, using Weighted median and MR Egger.

| **Exposure** | **Outcome** | **Method** | **B coefficient** | **Std Err** | **pval** |
| --- | --- | --- | --- | --- | --- |
| **Birth weight** | Atrial fibrillation | Weighted median | -0.219 | 0.044 | 5.58E-07 |
|  |  | MR Egger | -0.351 | 0.108 | 0.001 |
|  |  | intercept | 0.002 | 0.002 | 0.331 |
|  | Coronary artery disease | Weighted median | 0.172 | 0.037 | 2.37E-06 |
|  |  | MR Egger | 0.072 | 0.104 | 0.489 |
|  |  | intercept | 0.004 | 0.002 | 0.081 |
|  | Heart failure | Weighted median | 0.005 | 0.033 | 0.890 |
|  |  | MR Egger | -0.108 | 0.082 | 0.190 |
|  |  | intercept | 0.003 | 0.002 | 0.126 |
|  | Ischaemic stroke | Weighted median | 0.152 | 0.065 | 0.019 |
|  |  | MR Egger | -0.078 | 0.162 | 0.630 |
|  |  | intercept | 0.006 | 0.004 | 0.119 |
|  | LA Max Indexed | Weighted median | 0.006 | 0.046 | 0.898 |
|  |  | MR Egger | 0.048 | 0.090 | 0.597 |
|  |  | intercept | -0.001 | 0.002 | 0.512 |
|  | LATEF | Weighted median | 0.059 | 0.046 | 0.200 |
|  |  | MR Egger | 0.007 | 0.082 | 0.934 |
|  |  | intercept | 0.001 | 0.002 | 0.527 |
|  | LVESV Indexed | Weighted median | -0.134 | 0.049 | 0.006 |
|  |  | MR Egger | -0.061 | 0.105 | 0.562 |
|  |  | intercept | -0.003 | 0.003 | 0.323 |
|  | LVEDV Indexed | Weighted median | -0.147 | 0.048 | 0.002 |
|  |  | MR Egger | -0.061 | 0.107 | 0.568 |
|  |  | intercept | -0.003 | 0.003 | 0.228 |
|  | LVSV Indexed | Weighted median | -0.120 | 0.049 | 0.014 |
|  |  | MR Egger | -0.059 | 0.100 | 0.555 |
|  |  | intercept | -0.002 | 0.002 | 0.320 |
|  | LVEF | Weighted median | 0.057 | 0.048 | 0.234 |
|  |  | MR Egger | 0.021 | 0.092 | 0.819 |
|  |  | intercept | 0.001 | 0.002 | 0.642 |
|  | LV Mass Indexed | Weighted median | 0.116 | 0.040 | 0.004 |
|  |  | MR Egger | 0.176 | 0.093 | 0.059 |
|  |  | intercept | -0.001 | 0.002 | 0.631 |
|  | RA Max Indexed | Weighted median | -0.122 | 0.052 | 0.019 |
|  |  | MR Egger | -0.203 | 0.104 | 0.052 |
|  |  | intercept | 0.001 | 0.003 | 0.623 |
|  | RA Min Indexed | Weighted median | -0.159 | 0.051 | 0.002 |
|  |  | MR Egger | -0.240 | 0.101 | 0.018 |
|  |  | intercept | 0.002 | 0.002 | 0.423 |
|  | RA FAC | Weighted median | 0.119 | 0.047 | 0.011 |
|  |  | MR Egger | 0.146 | 0.086 | 0.095 |
|  |  | intercept | -0.002 | 0.002 | 0.454 |
|  | RVESV Indexed | Weighted median | -0.115 | 0.045 | 0.010 |
|  |  | MR Egger | -0.162 | 0.103 | 0.116 |
|  |  | intercept | 0.000 | 0.003 | 0.983 |
|  | RVEDV Indexed | Weighted median | -0.122 | 0.045 | 0.007 |
|  |  | MR Egger | -0.129 | 0.107 | 0.231 |
|  |  | intercept | -0.001 | 0.003 | 0.598 |
|  | RVSV Indexed | Weighted median | -0.114 | 0.051 | 0.027 |
|  |  | MR Egger | -0.047 | 0.101 | 0.645 |
|  |  | intercept | -0.003 | 0.002 | 0.298 |
|  | RVEF | Weighted median | 0.076 | 0.047 | 0.104 |
|  |  | MR Egger | 0.113 | 0.087 | 0.199 |
|  |  | intercept | -0.001 | 0.002 | 0.537 |
|  | Prox PA Diam Indexed | Weighted median | -0.017 | 0.053 | 0.748 |
|  |  | MR Egger | -0.096 | 0.113 | 0.398 |
|  |  | intercept | 0.003 | 0.003 | 0.167 |
|  | Asc Aorta Diam Indexed | Weighted median | -0.018 | 0.048 | 0.700 |
|  |  | MR Egger | 0.032 | 0.111 | 0.770 |
|  |  | intercept | 0.000 | 0.003 | 0.888 |
| **Fetal genetic influence on birth weight** | Atrial fibrillation | Weighted median | -0.221 | 0.062 | 0.000 |
|  |  | MR Egger | -0.282 | 0.138 | 0.044 |
|  |  | intercept | 0.001 | 0.003 | 0.777 |
|  | Coronary artery disease | Weighted median | 0.103 | 0.055 | 0.061 |
|  |  | MR Egger | 0.078 | 0.109 | 0.478 |
|  |  | intercept | 0.002 | 0.003 | 0.375 |
|  | Heart failure | Weighted median | -0.047 | 0.046 | 0.306 |
|  |  | MR Egger | -0.132 | 0.099 | 0.189 |
|  |  | intercept | 0.002 | 0.002 | 0.327 |
|  | Ischaemic stroke | Weighted median | 0.098 | 0.102 | 0.335 |
|  |  | MR Egger | -0.426 | 0.218 | 0.055 |
|  |  | intercept | 0.012 | 0.005 | 0.020 |
|  | LA Max Indexed | Weighted median | 0.028 | 0.063 | 0.660 |
|  |  | MR Egger | 0.073 | 0.100 | 0.470 |
|  |  | intercept | -0.002 | 0.003 | 0.334 |
|  | LATEF | Weighted median | 0.074 | 0.062 | 0.232 |
|  |  | MR Egger | -0.051 | 0.103 | 0.620 |
|  |  | intercept | 0.004 | 0.003 | 0.125 |
|  | LVESV Indexed | Weighted median | -0.144 | 0.062 | 0.021 |
|  |  | MR Egger | -0.075 | 0.112 | 0.508 |
|  |  | intercept | -0.001 | 0.003 | 0.661 |
|  | LVEDV Indexed | Weighted median | -0.147 | 0.066 | 0.027 |
|  |  | MR Egger | -0.109 | 0.120 | 0.371 |
|  |  | intercept | -0.001 | 0.003 | 0.691 |
|  | LVSV Indexed | Weighted median | -0.114 | 0.067 | 0.089 |
|  |  | MR Egger | -0.108 | 0.115 | 0.353 |
|  |  | intercept | -0.001 | 0.003 | 0.784 |
|  | LVEF | Weighted median | 0.048 | 0.061 | 0.431 |
|  |  | MR Egger | -0.011 | 0.093 | 0.903 |
|  |  | intercept | 0.001 | 0.002 | 0.632 |
|  | LV Mass Indexed | Weighted median | -0.120 | 0.065 | 0.065 |
|  |  | MR Egger | -0.167 | 0.136 | 0.222 |
|  |  | intercept | 0.001 | 0.003 | 0.772 |
|  | RA Max Indexed | Weighted median | -0.105 | 0.065 | 0.109 |
|  |  | MR Egger | -0.246 | 0.128 | 0.059 |
|  |  | intercept | 0.004 | 0.003 | 0.238 |
|  | RA Min Indexed | Weighted median | -0.150 | 0.066 | 0.022 |
|  |  | MR Egger | -0.315 | 0.122 | 0.013 |
|  |  | intercept | 0.006 | 0.003 | 0.070 |
|  | RA FAC | Weighted median | 0.069 | 0.071 | 0.330 |
|  |  | MR Egger | 0.232 | 0.102 | 0.027 |
|  |  | intercept | -0.005 | 0.003 | 0.048 |
|  | RVESV Indexed | Weighted median | -0.140 | 0.059 | 0.018 |
|  |  | MR Egger | -0.226 | 0.102 | 0.030 |
|  |  | intercept | 0.002 | 0.003 | 0.400 |
|  | RVEDV Indexed | Weighted median | -0.172 | 0.063 | 0.007 |
|  |  | MR Egger | -0.205 | 0.112 | 0.074 |
|  |  | intercept | 0.002 | 0.003 | 0.595 |
|  | RVSV Indexed | Weighted median | -0.114 | 0.067 | 0.087 |
|  |  | MR Egger | -0.111 | 0.108 | 0.309 |
|  |  | intercept | 0.000 | 0.003 | 0.997 |
|  | RVEF | Weighted median | 0.098 | 0.062 | 0.118 |
|  |  | MR Egger | 0.122 | 0.090 | 0.182 |
|  |  | intercept | -0.002 | 0.002 | 0.521 |
|  | Prox PA Diam Indexed | Weighted median | -0.024 | 0.068 | 0.728 |
|  |  | MR Egger | -0.023 | 0.130 | 0.863 |
|  |  | intercept | -0.006 | 0.007 | 0.378 |
|  | Asc Aorta Diam Indexed | Weighted median | -0.019 | 0.064 | 0.765 |
|  |  | MR Egger | 0.095 | 0.119 | 0.430 |
|  |  | intercept | -0.002 | 0.003 | 0.570 |
